# Micro-Macro Analysis of Complex Networks

**DOI:** 10.1371/journal.pone.0116670

**Published:** 2015-01-30

**Authors:** Massimo Marchiori, Lino Possamai

**Affiliations:** 1 Department of Mathematics, University of Padua, Padua, Italy; 2 Atomium Culture, Brussels, Belgium; Universidad de Zarazoga, SPAIN

## Abstract

Complex systems have attracted considerable interest because of their wide range of applications, and are often studied via a “classic” approach: study a specific system, find a complex network behind it, and analyze the corresponding properties. This simple methodology has produced a great deal of interesting results, but relies on an often implicit underlying assumption: the level of detail on which the system is observed. However, in many situations, physical or abstract, the level of detail can be one out of many, and might also depend on intrinsic limitations in viewing the data with a different level of abstraction or precision. So, a fundamental question arises: do properties of a network depend on its level of observability, or are they invariant? If there is a dependence, then an apparently correct network modeling could in fact just be a bad approximation of the true behavior of a complex system. In order to answer this question, we propose a novel micro-macro analysis of complex systems that quantitatively describes how the structure of complex networks varies as a function of the detail level. To this extent, we have developed a new telescopic algorithm that abstracts from the local properties of a system and reconstructs the original structure according to a fuzziness level. This way we can study what happens when passing from a fine level of detail (“micro”) to a different scale level (“macro”), and analyze the corresponding behavior in this transition, obtaining a deeper spectrum analysis. The obtained results show that many important properties are not universally invariant with respect to the level of detail, but instead strongly depend on the specific level on which a network is observed. Therefore, caution should be taken in every situation where a complex network is considered, if its context allows for different levels of observability.

## Introduction

Real world dynamical complex networks are non linear systems. This means that the full set of elements that interact pairwise (even in a trivial way) will result in a behavior that is often unpredictable. For a wide variety of such complex systems, the spatial informative component is crucial: for example, protein-to-protein networks, brain networks [1], transportation networks [2] [3], social networks [4], power grids [5], the Internet, companies networks [6], etc are all embedded in Euclidean space, and most interestingly, the space variable itself constraints their natural evolution. Being a structure embedded in space makes such network a physical object, that is the subject of observation. But as a physical object, every such network can be observed at various levels of precision. This means that our perception of a complex system also depends on the level of detail that we use in describing such system. This dependence is usually understood and given for granted, as the “classic” approach to study a complex system usually just focuses on extracting a network from a system, and then proceeding on the main part, that is the study of its properties. In this paper we instead focus on the neglected part, the starting point of all such analyses: the level of observability of a system. The main issue here is the following fundamental question: do properties of complex systems depend on the level of detail? And if so, to what extent? The complete answer to this question is of utmost importance in order to complete our knowledge of complex system, closing the circle of the properties and limitations of the “classic” approach. And conversely, when added as an evaluation parameter the observability level could help to characterize how it triggers environment changes.

On a more technical level, by varying the scale level of observability we focus our attention to the spatial characterization of networks, shedding light on how this can alter statistical measures of the graphs under study. We introduce a general framework of action, called *micro-macro* analysis as it concerns the study of the scale observable, in the large and in the small. This analysis is performed via a *micro-macro scaler*, that accomplish for the capability of distinguishing elements of the graph within a certain level of detail (represented by a fuzziness parameter), which represents the precision of observability, and therefore the scale level. The more a graph is close to the “micro” point of view (low fuzziness), the more precise the connectivity and nodes will be. Conversely, the more we go towards the “macro” level the more inaccurate and unclear the structure will become (high fuzziness). This accounts for the fact that many real systems are, for various reasons, just too difficult to determine in a totally precise way, and so the important question arises: do properties of a network depend on its level of observability, or are they invariant? If this is not the case, then an apparently correct network modeling could in fact just be a bad approximation of the true behavior of a complex system. By answering this fundamental question, we are able to state what are the properties that are safe to consider when abstracting networks, and conversely, which structures better preserves system attributes (for instance, are there differences in this regard between exponential and small world or scale-free networks?).

In particular, we investigate the variations of the statistical properties not only when the network detail is high (micro view) or low (macro view), but also in between these two extremes. The family of graphs calculated at these different intermediate resolution granularities forms a so-called *micro-macro spectrum*. We can therefore study what kind of transitions, if any, the properties of a complex system experience with respect to a scale variation. Relatedly, this analysis therefore allows to study the stability of a property, that is to say the variation of the property values in the micro-macro spectrum. Finding that many properties are unstable for a network during the abstraction process is therefore clear evidence that a single detail level analysis could suffer from incompleteness, and its results will be consequently dependent on the selected granularity.

**Fig 1:**
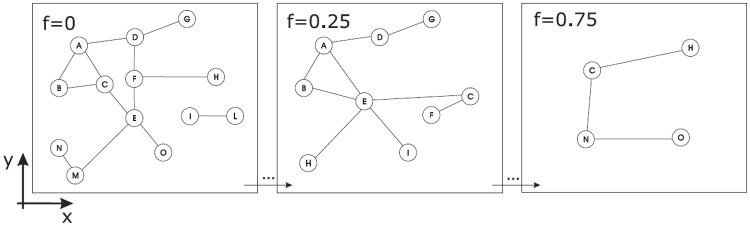
Example of micro-macro analysis. Example of micro-macro analysis obtained by increasing (abstraction process to the macro world) fuzziness *f*. When *f* = 0, no abstraction is applied whereas at increasing values of *f*, the network will be more obfuscated and the structure will be simpler. In the extreme situation when *f* is maximum, *f* = 1 (not displayed in the figure), the original network will be collapsed into a one node graph.

In order to concretely perform such analysis, we introduce a particular micro-macro scaler, that we call *telescopic*: the telescopic scaler defines scale abstraction levels with a spatial characterization modeled thru Euclidean zooming, in parallelism with the vision process typical of the human eyes. Every ocular observation is limited by the resolution power, i.e., the ability of the human eye to distinguish two points when placed at some distance from an observer. In the micro-macro scaling metaphor, the observation object is a graph and the points are nodes. The networks reconstruction is accomplished by tuning the distance parameter *f* (called equivalently fuzziness, representing the detail or granularity level) in order to virtually place a graph far or close to a fixed point of view. Small fuzziness values *f* (*f* → 0) yield clear networks with finer detail level, while big fuzziness values (*f* → 1) result in obfuscated networks, reassembling the abstraction process (see Fig. 1). Our telescopic scaler algorithm is able to handle both weighted and undirected graphs. Although more general in scope, we will employ the telescopic scaler with complex networks whose objects are endowed with classic Euclidean-spatial information, for example in the form of latitude and longitude nodes coordinates.

We applied our framework to a number of networks, both real world networks (such as rapid transportation systems like subways and airlines), and social-based networks). Indeed, we show how micro-macro analysis can provide great insights on what changes a network modeling (at a chosen level of detail) can introduce with respect to the real system, and correspondingly on what part of network analysis is potentially unsafe under certain modelings.

### Related work

Micro-macro analysis is based on the notion of scaling, and on the concept of being able to selectively give more importance to the macro world by washing out the micro details. This micro-macro dualism is at the basis of important works in physics: for instance, the pioneering work of Kadanoff [7], studying the statistical mechanics of critical scaling, introduced the “block spin” renormalization group, a transformation that renormalizes a magnetic system (the Ising model) by possibly aggregating 2×2 adjacent blocks of atoms in a square-lattice geometry. This concept of renormalization became extremely useful in physics, being applied also to other contexts and geometries like lattices (cf. [8] and [9]), and later on started to find applications directly in network theory, for instance when Newmann and Watts [10] used it in the lattice geometry that provided the first small-world model, and then Kim [11] applied the Kadanoff normalization group to a brain network formed by cubic cells (voxels, cf. [12]) embedded in a two-dimensional lattice geometry, assessing how network properties like degree exponent, clustering, assortativity and hierarchical structure vary by repeatedly applying renormalization. Another more recent application has been developed in [13], where renormalization is used to show that networks obtained from periodic and chaotic attractor bifurcation cascades have scale-invariant limiting forms.

Along with these achievements, Song et al. [14], employing the ideas of fractals and self-similarity under renormalization [15] [16], developed the concept of coarse graining, that reduces the size of a network by preserving the most representative properties at the cost of throwing away some finer details of the system. Coarse graining, inspired by the fact the structure of a fractal is similar no matter what length scale is chosen, tries to group together system units into specific box tilings (“box counting”) whose dimension determines the length scale at which the system is observed. The number of boxes *N*_*B*_ and the length scale *l* are shown to be related for various topological networks by the relationship *N*_*B*_ ∝ *l* ^−*d*^, with *d* the fractal dimension of the system. The concept of dimension of a network has then been object of further studies (see for instance [17]). Later, Radicchi et al. [18] [19] studied coarse graining in detail by considering multiple iterations of the renormalization process (what they call renormalization flows).

Our analysis further extends on the renormalization idea in two ways. First, we gather all these previous works under a unique micro-macro framework: the general concepts of micro-macro scaler and spectrum. Second, we introduce a novel specific scaler, the telescopic one, that enables to perform micro-macro analysis in a rather different way. The most important aspect is that the telescopic scaler allows to reason on completely general metric networks, and can therefore be used to study all those system that have a real-world spatial geometry of interaction, without artificially altering their dimensionality. Another important aspect is the physical grounding that lies behind the telescopic scaler: it is based on the notion of level of detail, and as such it has a very specific and well justified meaning related to observability.

Additionally, our analysis differs from the box-counting coarse graining of Song et al. [14] in the following points:

- The telescopic scaler considers Euclidean positions of the nodes, whereas the box-counting technique uses only the topological structure of the networks, throwing away useful information that comes with the spatial dimension of the vertices. Moreover the telescopic scaler can be more generally defined onto a metric space induced by a distance, and therefore also be applied to weighted networks, that describe system interactions in a much more precise level rather than a topological representation.
- In box counting, the number of boxes varies according to the length and fractal dimension. Vice versa, in the telescopic approach, the number of nodes belonging to boxes is not bounded: it depends on the spatial distribution of the nodes on the plane and on the fuzziness value. The maximum number of boxes is upper bounded and is inversely proportional to the fuzziness value (this concept will be extensively described in the following section).
- Box covering and telescopic analysis differ in the way they consider input and output graphs. In the former, output and input graphs are the same, in the sense that the input graph corresponds to the output of the previous step. Conversely, in the latter, the same graph is provided as input but different abstraction parameters will be applied at every step.
- The telescopic scaler is way more efficient, not requiring expensive resources to find specific box tilings: the tiling directly comes from the metric properties of the space, and can be very efficiently computed by using techniques drawn from spatial databases (see e.g. [20]).

Regarding Kim’s work [11], it is also different from ours in the following points, which make the telescopic scaler more general:

- In the telescopic scaler, the box dimension in the Euclidean space is not fixed and it is correlated to the spatial parameter *f*. This means that the number of nodes in a single box is variable and depends on the spatial distribution of the vertices across the plane.
- Our approach considers weights as, generally, distances between vertexes, whereas in Kim’s work weights represent the number of crossing edges between voxels.
- We do not remove edges to avoid the creation of complete networks as Kim did.
- The overall functional behavior of the (brain) network that Kim consider in his paper is fundamentally different from ours. In fact, he does not consider the actual path of voxels through which the biochemical signal transfers.
- With a micro-macro scaler (like the telescopic one) we can directly obtain a specific scale level of detail by starting from the original graph and using a fuzziness value indicating the level of abstraction applied. Conversely, Kim’s approach is similar to the original work of Song et al. [14] in which the output’s graph at step *t* is used as input at the *t* + 1 iteration.

## Materials and Methods

The aim of this section is (i) to describe our new micro-macro analysis, using the *telescopic* algorithm, that is capable of abstracting networks at various granularities and (ii) to assess whether the statistical properties usually employed in complex networks analysis are affected by the multidimensional network analysis itself. We start by giving the necessary background definitions, go on by introducing the notion of micro-macro scaler and the specific telescopic one, and then proceed by performing micro-macro analysis on several real-world complex systems.

### Graph Theory

A (*simple*) graph G is a pair (*V*, *E*) where *V* = {*u*_1_, *u*_2_, …, *u*_*n*_}, ∣*V*∣ = *n* is a finite set of vertices and *E* ⊆ *V* × *V*, *E* = {(*u*_*i*_, *u*_*j*_), *i* ≠ *j*}, ∣*E*∣ = *m* is the set of edges that links couples of nodes. These graphs are called *topological*. A graph *G* can be represented by a *n* × *n* adjacency matrix *A* with entries *a*_*ij*_ = 1 when (*u*_*i*_, *u*_*j*_) ∈ *E*, *a*_*ij*_ = 0 otherwise. *a*_*ii*_ = 1 denotes self loops. A weighted graph is defined as *G* = (*V*, *E*, *w*) where *w* is a function that assigns real values to edges. In undirected graphs, (*u*, *v*) ∈ *E* ⇔ (*v*, *u*) ∈ *E* and the adjacency matrix *A* will be symmetric (with respect to its diagonal, that consists of all zeros if self loops are not allowed). Conversely, in directed graphs, or *digraphs*, each edge (sometimes referred as arc or link) has an orientation, so (*u*_*i*_, *u*_*j*_) ≠ (*u*_*j*_, *u*_*i*_).

Metrical (also known as spatial) graphs extend weighted graphs as they are spatially embedded, that is, every node exists in a Euclidean coordinates’ space. Specifically, *G* = (*V*, *E*, *C*, *w*) where *C* = {(*x*_1_, *y*_1_), (*x*_2_, *y*_2_)⋯, (*x*_*n*_, *y*_*n*_)} is the set of node coordinates (e.g., a spatial position in terms of latitude and longitude) and the function *w* might assigns, for instance, Euclidean distances between nodes.

**Fig 2:**
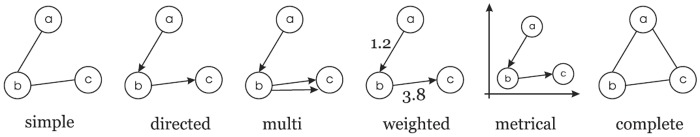
Graph types. Examples of different graph types.

*Multigraphs* are generalized graphs in which the same couple of nodes might be connected by more than one edge. Even though many real world complex systems could be represented by multigraphs, in many occasions these networks are transformed into weighted graphs in such a way that the number of edges connecting two nodes is reflected in the edge weight of the new graph (see Fig. 2 for a summary of of graph classes).

Given two graphs *G*_1_ = (*V*_1_, *E*_1_) and *G* = (*V*_1_, *E*_1_), a *graph homomorphism* is a function *ϕ*:*V*_1_ → *V*_2_ preserving edges, i.e., such that ∀(*i*, *j*) ∈ *E*_1_.(*ϕ*(*i*), *ϕ*(*j*)) ∈ *E*_2_. A *graph epimorphism* is a surjective graph homomorphism.

Given a graph *G*, we denote with ∣*G*∣_*V*_ the cardinality of its vertexes, and with ∣*G*∣_*V*_ the cardinality of its edges.

A *path* is a non empty graph *P* = (*V*, *E*) in the form of

$$V=\{u_{0},u_{1},\ldots ,u_{k}\}\;E=\{(u_{0},u_{1}),(u_{1},u_{2}),\ldots ,(u_{k-1},u_{k})\};$$

*simple paths* are those in which all vertices *u*_*i*_ are distinct. The number of edges in a path determines its length and a path of length *k* is defined as *P* ^*k*^. A path from *a* to *b* of length *k* is a path *P* ^*k*^ in which *u*_0_ = *a* and *u*_*k*_ = *b*. A graph *G* is *connected* if for each *u*_*i*_, *u*_*j*_ ∈ *V*, *i* ≠ *j*, there exists a simple path from *u*_*i*_ to *u*_*j*_ (denoted as $u_{i}⇝u_{j}$). In order to simplify notations, in this paper we equivalently specify nodes as *i* or *u*_*i*_.

An important graph property is the *shortest path* between two vertices, *d*_*ij*_ (also known as *geodesic*). The definition of shortest path depends on the class of graphs we are dealing with. In simple graphs, the shortest path between nodes *i* and *j* represents the minimum number of traversed nodes (hops) to reach *j* from *i*. If the graph is connected, it is natural to observe that ∀*i*, *j* *d*_*ij*_ ≥ 1, and *d*_*ij*_ = 1 if node *i* is directly connected to node *j*. If there are no paths between *i* and *j* then *d*_*ij*_ = ∞. Indeed, in weighted graphs, the shortest path is calculated taking into account the weights on edges such that *d*_*ij*_ = min{*w*_*p*_∣*p* is a path between *i* and *j*} where

$$w_{p}=\sum_{e\in E(p)}w(e)$$

is the sum of edge weights along path *p*.

The *diameter* *D* of graphs is usually defined as the maximum *d*_*ij*_ between every couple of nodes. However, since *d*_*ij*_ depends on the graph type, *D* could also have the following meanings: the number of hops that separates two vertices, the maximum shortest weighted path or the maximum Euclidean distance between the farthest nodes, without considering the underlying topological structure (in this case we refer to *physical diameter*).

The *degree* of a node *u* in a graph corresponds to the cardinality of the set *N*(*u*) = {*v* ∈ *V* ∣ (*u*, *v*) ∈ *E*} = *deg*(*u*) = *k*_*u*_ and ∑_*u* ∈ *V*_ *deg*(*u*) = 2∣*E*∣. When *deg*(*u*) = 0, then *u* is said to be *isolated*. In directed graphs, it is customary to split node degree into inbound *k* ^*in*^ and outbound *k* ^*out*^ degree. Indeed, the degree distribution *P*(*k*) that corresponds to the probability of having a node with degree *k*, has to be split into two parts, inbound *P* ^*in*^(*k*) and outbound *P* ^*out*^(*k*) degree distribution.

The *average degree* of a graph ⟨*k*⟩ (or *k*_*mean*_) is $1/n\sum_{i=1}^{n}k_{i}$ and the strength [21] *s*_*i*_ of node *i* is the sum of the weights of the edges incident on *i*, *s*_*i*_ = ∑_*j*_ *w*_*ij*_. In directed graphs, the strength can be split relative to the edges directions, reflecting the total inbound and outbound weight, as for the node degree and the degree distribution.

A graph *G* is *complete* if for each *i*, *j* ∈ *V* (*i* ≠ *j*) ⇒ (*i*, *j*) ∈ *E*. In the literature, complete graphs are usually denoted as *K*_*n*_, with *n* representing the total number of nodes and $|E|=\frac{n(n-1)}{2}$ if the graph is undirected, *n*(*n* − 1) otherwise. Recent experiments showed that those graphs are rare to find in nature mainly because of the inherent high cost of creation and maintaining such a redundant structure. Think, for instance, of having a telephone network in which there exist direct connections between every user. This class of networks are usually used in ideal contexts or as normalizing factor in formulas (see the next subsection Statistical Properties).

A graph *T* is a *subgraph* of a graph *G*, denoted by *T* ⊆ *G*, when *V*_*T*_ ⊆ *V*_*G*_ and *E*_*T*_ ⊆ *E*_*G*_ holds. *V*_*T*_ and *V*_*G*_ are the set of nodes of *G* and *V* respectively. A graph *T* ⊆ *G* is said to be *induced* when *E*_*T*_ = {(*u*_*i*_, *u*_*j*_) ∈ *E*_*G*_∣*u*_*i*_ ∈ *V*_*T*_, *u*_*j*_ ∈ *V*_*T*_}.

The previous definitions are only a subset of all concepts and ideas that have been developed in the graph theory literature. For interested readers, we refer to Diestel’s [22] book.

### Network Properties

Here, we present an overview of the most important network properties that will be later analyzed in a micro-macro perspective.

Watts and Strogatz [5] proposed two effective and intuitive metrics, namely the characteristic path length *L* and the clustering coefficient *C*. The first measures the typical separation between two vertices in a graph (a global quantitative measure of graphs), whereas the second measures the cliquishness of a typical neighborhood (a local property) [23]. More formally, the former is calculated as

$$L(G)=\frac{1}{n(n-1)}\sum_{i\neq j\in V}d_{ij}.$$

Since real world networks might have disconnected subgraphs (for example Escherichia coli [24] or some protein to protein networks [25]), network scientists usually restrict their study to the largest connected component (LCC), in which *d*_*ij*_ < ∞ for each (*u*_*i*_, *u*_*j*_) ∈ *E*. The results, in order to be significant have to be calculated on a big LCC, i.e., the fraction of nodes that belongs to it must be very high so to be a good representative of the original network.

Vice versa, the clustering coefficient *C* is formally described as the mean of all *C*_*i*_’s, namely:

$$C(G)=\frac{1}{n}\sum_{i\in V}C_{i}\;C_{i}=\frac{E[G_{i}]}{k_{i}(k_{i}-1)/2}$$

where *C*_*i*_ is the fraction between the numbers of edges of the subgraph *G*_*i*_ over the total number of edges of *K*_*i*_. Subgraph *G*_*i*_ is the graph of the neighbors of node *i* (*i* excluded).

Latora and Marchiori [26] developed a set of metrics, based on the concept of efficiency *ε*, that allow considering both connected and disconnected graphs. They define global efficiency of a graph *G* as:

$$E_{glob}(G)=\frac{\sum_{i\neq j\in V}\epsilon_{ij}}{n(n-1)}=\frac{1}{n(n-1)}\sum_{i\neq j\in G}\frac{1}{d_{ij}}$$

as the average of efficiency *ϵ*_*ij*_ of the graph. Here, they assumed that efficiency *ϵ*_*ij*_ and distance *d*_*ij*_ are inversely proportional. However, other relationships might be used (instead of *d*_*ij*_), especially when justified by a more specific knowledge about the system. Nevertheless, *d*_*ij*_ will have different meanings in weighted and unweighted networks. In the first case, it corresponds to the number of hops between two nodes in the shortest path (*topological efficiency*) whereas in the second one is the sum of all edge weights in the shortest path (*metrical efficiency*). Global efficiency, as defined above, ranges from 0 to +∞. In practical applications, it is convenient to normalize it by the ideal network *K*_*n*_, namely *E*_*glob*_(*G*)/*E*_*glob*_(*K*_*n*_) such as 0 ≤ *E*_*glob*_(*G*) ≤ 1, therefore it can be used to compare efficiency of different graphs.

On the other side of the same measure, the efficiency can be used to evaluate any subgraph of G, and therefore to characterize the local properties of a network as the following:

$$E_{loc}(G)=\frac{1}{N}\sum_{i\in V}\frac{E_{glob}(G_{i})}{E_{glob}(G_{i}^{ideal})}$$

that is merely the average of the global efficiency applied to each subgraph *G*_*i*_, normalized by the referring ideal graph $G_{i}^{ideal}$.

Moreover, the same authors proposed a statistical property that accounts for the *cost* of a network, defined as:

$$Cost(G)=\frac{2m}{n\cdot (n-1)},\;Cost(G)=\frac{\sum_{i\neq j\in G}a_{ij\;}\gamma (d_{ij})}{\sum_{i\neq j\in G}\gamma (d_{ij})}$$

The leftmost formula is used in unweighted networks and is usually known as *density* whereas the rightmost accounts for weighted networks where *a*_*ij*_ is an element of the graph adjacency matrix *A* and *γ* is the cost evaluator function which calculates the cost needed to build up a connection with a given distance (length) *d*_*ij*_.

In many real world networks the degree distribution does not follow a bell curve (that for instance characterizes the frequency of humans heights), but instead does follow a power law, i.e. *P*(*k*) ∼ *c*⋅*k* ^−*γ*^ where *c* is a constant and *γ* is a positive exponent that empirically varies between two and three. Having a *P*(*k*) that has a decaying tail in the power law means that the vast majority of nodes have low degree and that there exist few nodes, the so-called *hubs*, that have an extremely high connectivity. Such networks have been named *scale-free* [27], because power-laws have the property of having the same functional form at all scales. Nevertheless, when working with real networks it may happen that the data have a rather strong intrinsic noise due to the finiteness of the sampling. Therefore, when the system size is small and the degree distribution *P*(*k*) is heavy-tailed, it is sometimes advisable [28] to measure the *cumulative degree distribution* $P_{cum}(k)=\sum_{k^{\prime }=k}^{\infty }P(k^{\prime })$. Indeed, when summing up the original distribution *P*(*k*), the statistical fluctuations generally present in the tails of the distribution will be smoothed. Consequently the exponent *γ* of *P*(*k*) ∼ *k* ^−*γ*^ can be obtained from *P*_*cum*_(*k*) as one plus the slope of *P*_*cum*_(*k*) in a log-log plot, i.e., *γ* = 1 + *γ*_*cum*_.

Another fundamental property of networks is the *degree-degree correlation* (also known as network *assortativity*). This feature is extremely important in the resilience of networks [29] [30] but it also has a strong impact on the network dynamical properties, such as spreading processes. In *assortative* networks, most edges connect nodes that exhibit similar degrees (nodes aristocracy). On the other hand, *disassortative* networks are such that high-degree nodes are connected to low-degree nodes.

More analytically, the network correlation *k*_*nn*_ between vertices is calculated as *k*_*nn*_(*k*) = ∑_*k*^′^_ *k* ^′^ *P*(*k* ^′^∣*k*) where *P*(*k* ^′^∣*k*) is the conditional probability that a node with degree *k* is connected to a node with degree *k* ^′^. If there is no degree correlation, the formula simplifies to *k*_*nn*_(*k*) = ⟨*k* ^2^⟩/⟨*k*⟩, i.e. is independent of *k*. Positively correlated graphs are classified as assortative if *k*_*nn*_ is an increasing function of *k*, whereas they are referred to disassortative when *k*_*nn*_(*k*) is a decreasing function of *k* [31]. Degree correlations are usually quantified by reporting the numerical value of the slope of *k*_*nn*_(*k*) as a function of *k* or by calculating the Pearson correlation coefficient of the degrees at either ends of a link [32]. ER graphs are, by definition, uncorrelated graphs, since the edges are connected to nodes regardless of their degree. Consequently, the assortative-mixing value is neutral (zero). This holds also for the preferential attachment model proposed by Barabási-Albert [33].

For a survey of all the previous network statistical properties and more we refer the reader to [34].

### Micro-Macro Analysis

We have already described the intuition behind micro-macro analysis. We can now be more formal and define the main tool by which such analysis can be performed.

A *micro-macro scaler* is a function *μ* that takes as inputs a graph *G* and a fuzziness level *f* (a real in the range [0, 1]), and gives back another graph *G*′_*f*_ together with a graph epimorphism *ϕ*_*f*_ : *G* → *G* ^′^, such that:

- *μ*(*G*, 0) = (*G*, 1_*G*_) (where 1_*G*_ is the identity map on *G*)
- *μ*(*G*, 1) = (*G* ^′^, *ϕ*_1_) with ∣*G* ^′^∣_*V*_ = 1
- if *f* < *f* ^′^ then ∣*G*′_*f*_∣_*V*_ ≥ ∣*G*′_*f*′_∣_*V*_
- if *f* < *f* ^′^ then ∣*G*′_*f*_∣_*E*_ ≥ ∣*G*′_*f*′_∣_*E*_

That is to say, a micro-macro scaler allows, for any complex network *G*, and a set level of detail *f*, to obtain its corresponding abstraction *G*′_*f*_, together with a precise correspondence given by the epimorphism *ϕ*_*f*_. The normalized range [0, 1] of *f* represents the values from 0 (micro level, finer level of details) to 1 (macro level, worst level of detail), formalized by the two conditions about the result for *μ*(*G*, 0) and *μ*(*G*, 1). The last two conditions state that a micro-macro scaler is anti-monotonic w.r.t. the number of edges and vertexes, meaning that the more we go from the micro to the macro level, the more detail we lose.

Given a micro-macro scaler *μ*, and a resolution level *r* (an integer ≥ 1), we can define for any starting graph *G* its so-called *micro-macro spectrum*, that is to say the whole family of networks $S_{G}=\{G_{0},G_{1},G_{2},\ldots ,G_{r}\}$ where *G*_*i*_ = *μ*(*G*, *i*/*r*). Given a resolution level, the spectrum therefore describes the whole behavior of a network when passing from the micro to the macro world (and as such, a spectrum can be then subsetted so to select different transition views).

### The Telescopic Scaler

Having introduced the general tools that make micro-macro analysis possible, we now go on by producing a suitable instance of a micro-macro scaler: the *telescopic scaler*. The telescopic scaler uses an algorithm that resembles the resolution power of human eyes, i.e., the ability to distinguish two points when placed at some *distance* from an observer. This way, the distance (proximity) corresponds to the level of fuzziness perceived by an observer: the more an object is far away from the viewer the more obfuscated it will be. The observed objects in our context are networks, and nodes are points in the metaphor of the human eyes resolution power. For instance, parts of a network that are close to a virtual observer are clearly distinguishable and therefore are characterized by a finer level of detail (“micro” level). Conversely, in networks far away from the point of view, the nodes will be obfuscated and the overall structure will be simpler than the original one (mimicking an abstraction process and the corresponding level of observability, going towards a “macro” level). In the rest of this paper, we interchangeably use fuzziness, distance, details or resolution level as synonyms of granularity with which a network has been described.

The networks we consider are defined as weighted and undirected graphs *G* = (*V*, *E*, *C*). For simplicity, we assume that the latitude and longitude coordinates of the nodes (*x*_*i*_, *y*_*i*_)_*i* = 1,⋯*n*_ (see the subsection Graph Theory), are normalized in [0, 1]. Edge weights are real normalized distances between nodes.

More precisely, we define the telescopic function as *t*:(*G* × *f*) → *G* ^′^ that takes a graph *G* and a value of fuzziness *f* as parameters and return the abstracted graph *G* ^′^. In this way, by applying repeatedly the function *t* with different values of *f* we obtain a micro-macro spectrum that is formed by a family of networks $S_{G}=\{G_{1},G_{2},\ldots ,G_{k}\}$ where *G*_*i*_ = *t*(*G*, *f*_*i*_), *k* sets the spectrum resolution and *f*_*i*_ is the fuzziness value of the *i* ^*th*^ step. A small value of *f*_*i*_ leads to clear view of networks and thus the resulting graphs *G*_*i*_ will have the finer detail level. Conversely, when *f*_*i*_ is big (*f* → 1) the view will be obfuscated and in the limit when *f*_*i*_ = 1 only one node will belong to the outcome network.

Network abstraction is accomplished by two distinct phases. The first one deals with creating nodes in *G*_*i*_ while the second defines the topological structure. Intuitively, nodes in *G*_*i*_ are the result of collapsing nodes in *G* that are close each other, hence not clearly distinguishable from an observer. The number of nodes that has to be collapsed obviously depends on *f* and on their spatial distribution on the plane.

**Fig 3:**
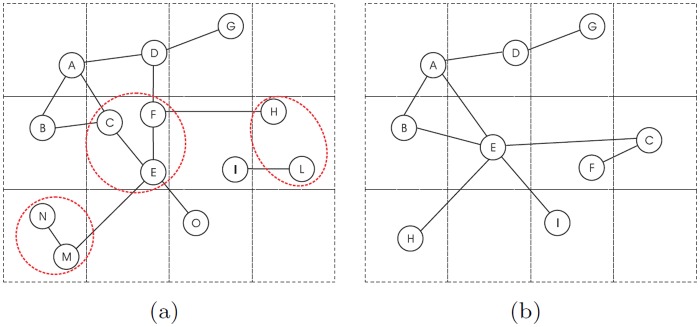
One-step abstraction process. One-step application of the abstraction process to a small graph. (a) Original graph *G*. Red (dashed) circles identify the group of nodes that will be merged together. (b) Output graph *G*_*i*_ in which nodes *c*, *f*, *e*, *h*, *l* and *n*, *m* are collapsed into new nodes *e*, *c*, *h* ∈ *V*_*i*_ respectively. Coordinates are the barycenter of collapsed nodes. Three edges are then removed because they connect the collapsed nodes: (*n*, *m*), (*c*, *e*), and (*f*, *e*).

**Fig 4:**
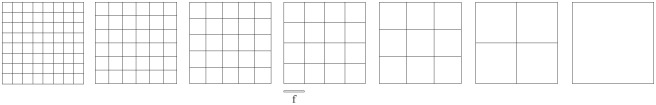
Sequence of box covering iteration for telescopic analysis. Example of grids applied on top of networks as a function of fuzziness. Leftmost grid has low fuzziness *f* = 0.125 whereas the rightmost has *f* = 1. The granularity of the spectrum in this example is equal to 7. In this paper, we only consider linear increase of *f*.

More technically, the process by which nodes in *G*_*i*_ are created is based on placing a virtual grid on top of *G* (see Fig. 3). This grid is formed by a set of square boxes whose spatial dimensions corresponds to the fuzziness *f* (see Fig. 3 and 4). Since we assumed that 0 ≤ *f* ≤ 1 and coordinates 0 ≤ *x*_*i*_, *y*_*i*_ ≤ 1, the total number of square boxes will be *N*_*B*_ = *f* ^−2^.

**Fig 5:**
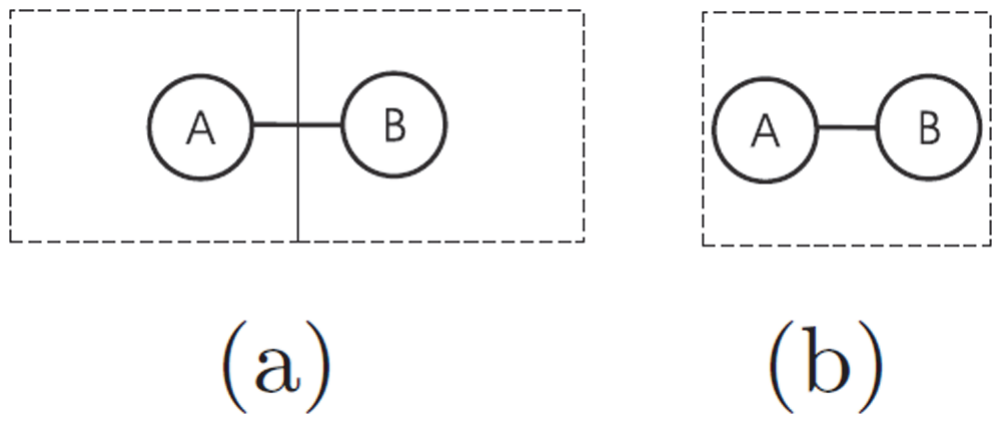
Box covering issue. Grid displacement issue when the distance between two nodes is less than fuzziness value. Wrong (a) and correct (b) grid displacement.

All nodes of *G* that belong to the same square cell are collapsed into a new node in *G*_*i*_ and new coordinates will be the barycenter of the collapsed nodes. The maximum number of nodes in *G*_*i*_ with fuzziness *f* is bounded to ∣*V*_*i*_∣_*f*_ ≤ *f* ^−2^ (maximum one node per box). This procedure aims at grouping nodes that are far by almost *f* units (eventually $f\sqrt{2}$ if the two points are at the extremes of the diagonal). However, the limitation of this algorithm is that not all nodes that are close each other by almost *f* units will be collapsed. This circumstance occurs when the grid fall in between neighbors’ nodes as Fig. 5(a) shows, whereas another grid placement like in Fig. 5(b) would have produced an equally sensible abstraction. For taking into account this issue, we applied a random grid shift that attenuates the bias introduced by grid displacement (see results in the Discussion section) and take averaged results of the statistical properties considered.

In the second phase, once vertices of *G*_*i*_ are defined, we re-establish the network connectivity. Here we adopt the most straightforward rule that preserves network structure: if two clusters of collapsed nodes of *G* are connected by at least one path, then in *G*_*i*_ the two representative nodes will be connected. Let’s define this concept in more detail using the notation presented above. Let Γ_*i*_ a set of nodes that belongs to box *i* and

$$g_{ij}=\{(k,m)\in E\;|\;k\in \Gamma_{i},\;m\in \Gamma_{j}\;\text{with}\;i\neq j\}$$

a set of edges whose source and target nodes belong to *i* and *j* box respectively. An edge (*u*, *v*) ∈ *E*_*i*_ ⇔ ∣*g*_*uv*_∣ > 0.

**Fig 6:**
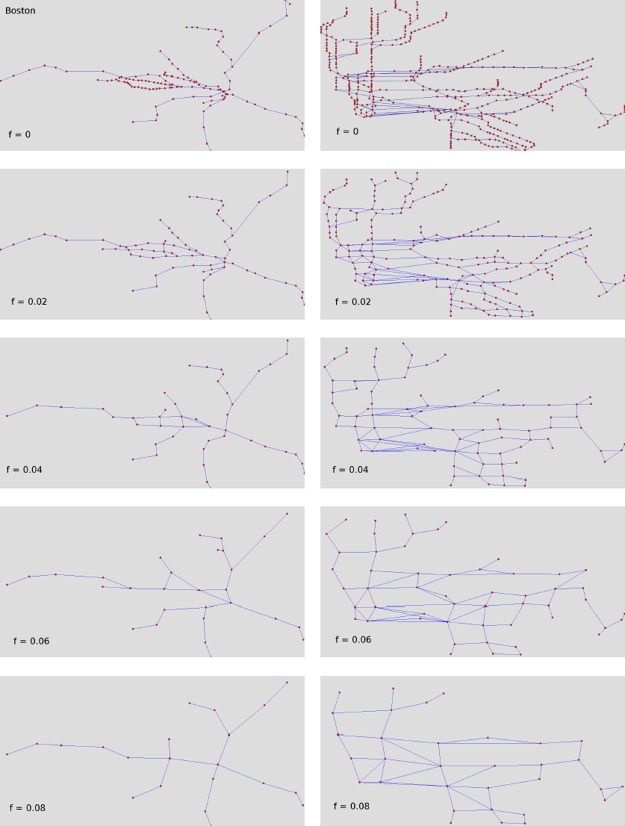
Example of box covering on real networks. How Boston (leftmost panels) and New York (rightmost panels) subway networks vary in the micro-macro spectrum according to increasing values of fuzziness. From the panel, it is clear that the spatial structure of the systems remain relatively unchanged in the first steps of the abstraction process.

Fig. 6 shows an example of application of telescopic analysis to the Boston and New York subway networks.

## Results

In this section, we report our experimental analysis. The telescopic algorithm was implemented as a C module and used in a Python script. All the experiments were conducted on three Linux machines equipped with i5 Intel processors at 3.2 Ghz and 8Gb of RAM.

### Datasets

We conducted experiments on several datasets, composed by *rapid transportation networks*, and by *online social networks*. We decided to consider subway networks because they are a fundamental element of mass transportation in urban areas and important means of cost reduction in transportation. Indeed, in the literature there exist some important network studies [35] [36] [37] that characterized the most important subway networks. The networks we used in our experiments are the Paris Métro, the Milan Metropolitana, and the New York and Boston Subways. The Paris Métro, one of the densest and busiest networks in the world, has sixteen lines and the first line opened in 1900. It has 295 stations connected by 346 rail connections. The Milan Metropolitana, the smallest subway network we will consider, is the biggest rapid transit system in Italy, opened in 1964. It has 81 stations and 80 rail connections. The New York subway is the most extensive rapid transportation system in the world by number of stations. It has 487 stations and 439 connections and opened in 1904. Finally, the Boston subway consisting of 124 stations and 125 connections had the first subway line opened in the United States in 1897.

Each node stands for a station, edges for direct railway connection between stations. Networks are created collecting latitude and longitude coordinates about station locations and converting them into *x*, *y* coordinates using Miller cylindrical projection [38] (Mercator projection might be another technique to use). We finally normalize them in such a way that every couple (*x*_*i*_, *y*_*i*_) ∈ [0, 1]. We also consider the US airline transportation system in which nodes represent airports and edges are non-stop flights. The US airline network (taken from [39]) has 235 airports and 1296 non-stop flights.

To investigate the effect of this novel analysis to other than transportation networks, we also consider online social networks. In particular, we analyze the VirtualTourist [40] social system. VirtualTourist (in the following abbreviated as VT) is an on-line tourist guide in which users share their travel experiences, suggest and review hotels, write comments and opinions on VT forums, find a place to visit, share photos and videos: it is a community of people that love traveling around the world. Users can meet new people and create a network of virtual friendships, making the VirtualTourist system a hybrid between Tripadvisor and Facebook.

The VirtualTourist social network is explored by web harvesting [41] all the publicly available profiles, and for each anonymized user collecting the following attributes: gender, birth date, subscription date and living location. We filter out users with empty location or unreliable fields (for example those whose format is not compliant).

Since VT locations span more than 150 countries, we decided to select only those countries with the highest number of users such as Australia, India, Italy, the Netherlands and the United Kingdom, and analyze them individually.

In order to obtain a spatial complex network, we decided to select the cities as the observable level (which is in fact the most precise level of detail available by using these public data about the users).

**Fig 7:**
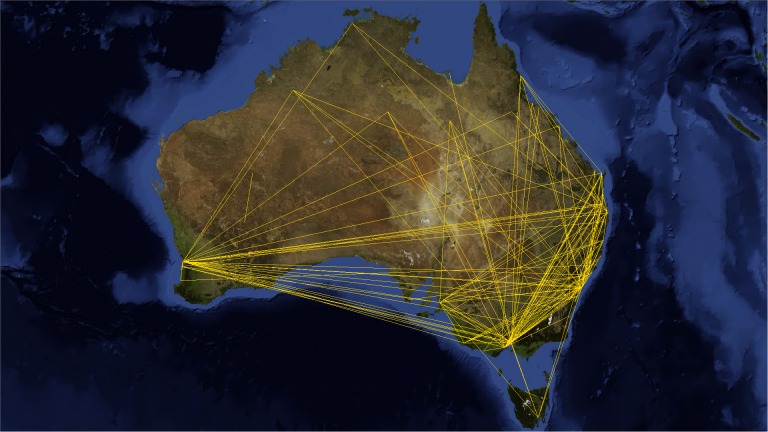
Australia’s city-based online social network. The online social networks of Australia created from its VirtualTourist online community. Lines (yellow) represent edges of the network connecting cities that share at least one friend. Background satellite image TIROS-3 courtesy of NASA (the U.S. National Aeronautics and Space Administration) and NOAA (the U.S. National Oceanic and Atmospheric Administration).

**Fig 8:**
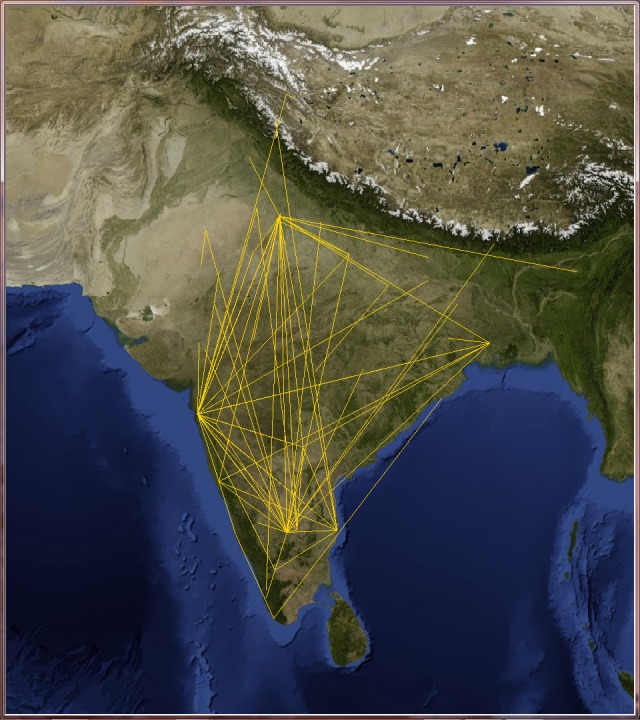
India’s city-based online social network. The online social networks of India created from its VirtualTourist online community. Lines (yellow) represent edges of the network connecting cities that share at least one friend. Background satellite image TIROS-3 courtesy of NASA (the U.S. National Aeronautics and Space Administration) and NOAA (the U.S. National Oceanic and Atmospheric Administration).

**Fig 9:**
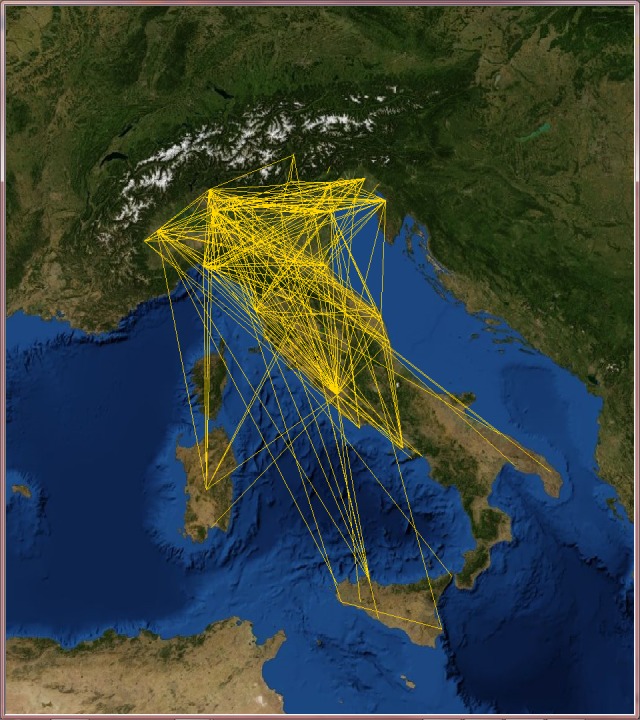
Italy’s city-based online social network. The online social networks of Italy created from its VirtualTourist online community. Lines (yellow) represent edges of the network connecting cities that share at least one friend. Background satellite image TIROS-3 courtesy of NASA (the U.S. National Aeronautics and Space Administration) and NOAA (the U.S. National Oceanic and Atmospheric Administration).

**Fig 10:**
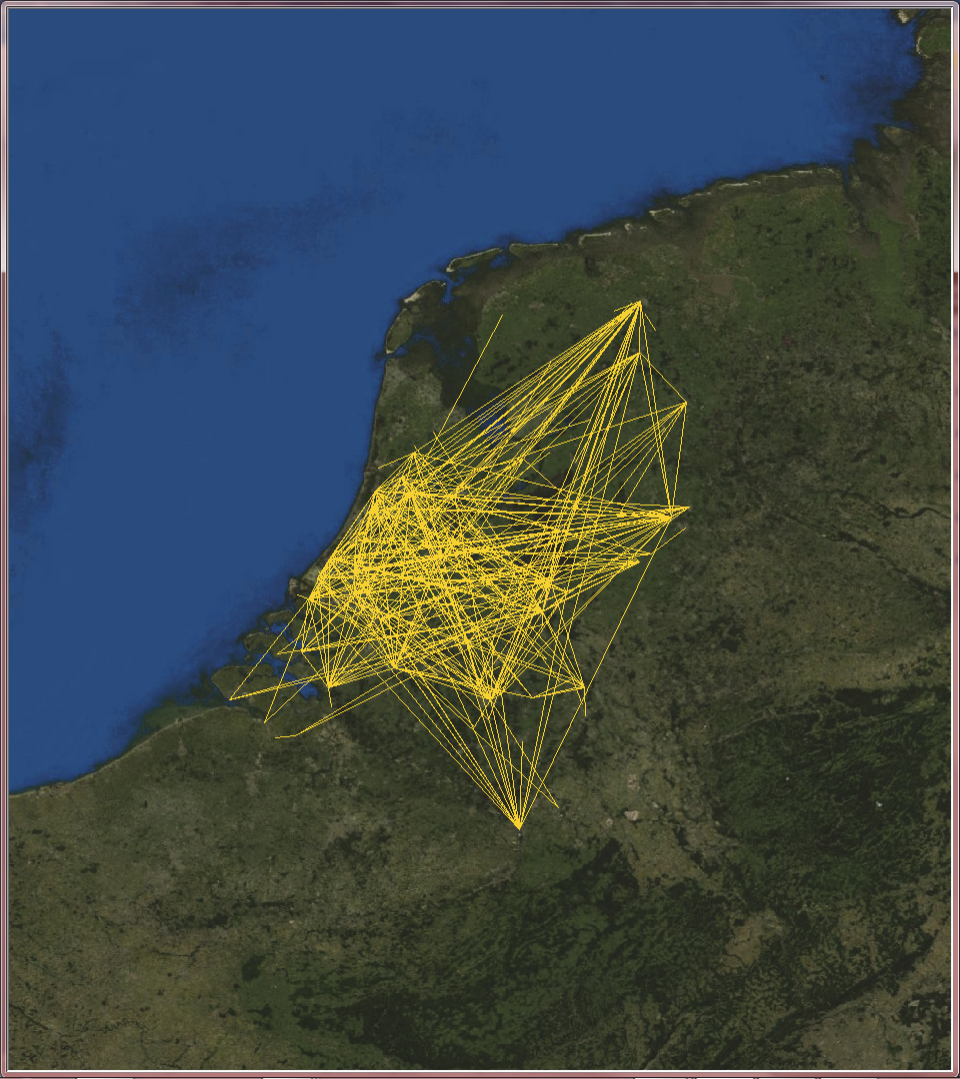
The Netherlands’s city-based online social network. The online social networks of the Netherlands created from its VirtualTourist online community. Lines (yellow) represent edges of the network connecting cities that share at least one friend. Background satellite image TIROS-3 courtesy of NASA (the U.S. National Aeronautics and Space Administration) and NOAA (the U.S. National Oceanic and Atmospheric Administration).

**Fig 11:**
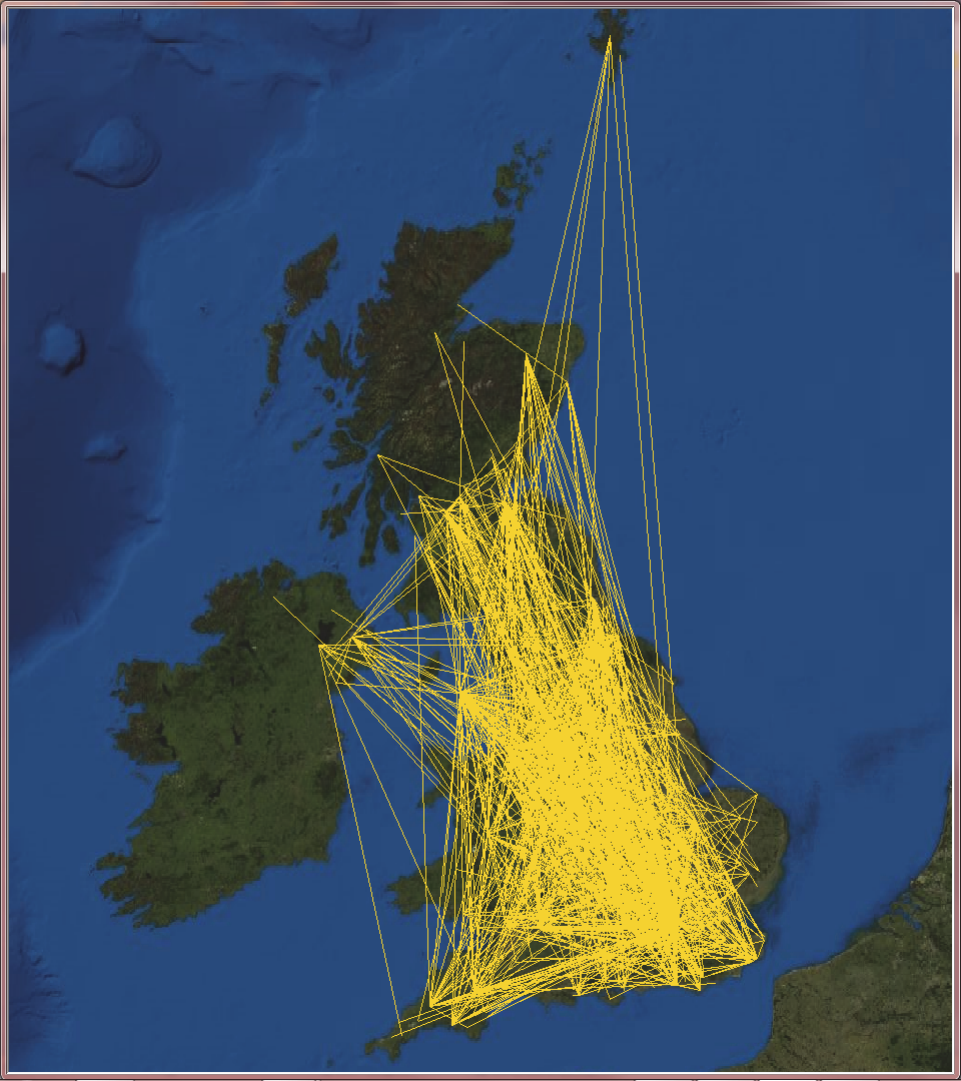
United Kingdom’s city-based online social network. The online social networks of the United Kingdom created from its VirtualTourist online community. Lines (yellow) represent edges of the network connecting cities that share at least one friend. Background satellite image TIROS-3 courtesy of NASA (the U.S. National Aeronautics and Space Administration) and NOAA (the U.S. National Oceanic and Atmospheric Administration).

Applying the telescopic algorithm to these networks may induce an unexpected increase in the number of collapsed nodes starting immediately at small values of *f*. This is caused by the presence of many users at the same location (for example when they live in big cities). In order to overcome this issue, we decided to transform these online social networks into city-based online social networks, where nodes stands for cities (in which lives at least one VT user) and links express friendship relations between users of those cities. These networks now describe friendship relations at the level of cities instead of the users. The GPS coordinates of the cities were gathered from the Geonames open source web service [42] and the edge weights are the Euclidean normalized distance between cities (see Figs. 7, 8, 9, 10 and 11). The network of Australia has 76 cities and 183 links (social ties), the Netherlands has 106 cities and 340 links, India has 46 cities and 81 links, Italy has 85 cities and 270 links, and finally the United Kingdom has 446 cities and 1322 links. Both transportation and city-based online social networks are undirected because people can move either in both directions of the transportation line and friendships relations in VT are bidirectional. Table 1 reports statistics of the datasets we used in this section of the paper. We calculated the most important statistical properties such as the number of nodes *n*, edges *m*, maximum degree *k*_*max*_, average degree ⟨*k*⟩, standard deviation of the degree *σ*_*k*_, degree correlation *ρ*, diameters, local and global efficiencies and costs (see the subsection Statistical Properties for the definitions).

**Table 1:** Statistical features of transportation and city-based online social networks.

	Boston	Milan	New York	Paris	US airline	Social IT	Social AU	Social NL	Social IN	Social UK
*n*	124	80	439	295	235	85	76	106	46	446
*m*	125	81	487	346	1296	270	183	340	81	1322
*k*_*max*_	4	4	8	8	130	40	31	28	22	188
⟨*k*⟩	2.02	2.03	2.22	2.35	11.03	6.35	4.82	6.42	3.52	5.93
*σ*_*k*_	0.54	0.57	0.81	1.06	17.98	7.82	6.81	7.68	4.71	12.66
*ρ*	0.32	-0.08	0.14	-0.07	-0.36	-0.26	-0.43	-0.07	-0.44	-0.19
*D*_*t*_	43	34	68	38	4	5	5	6	6	7
*D*_*p*_	1.08	1.12	1.15	1.17	1.26	1.08	1.01	1.07	1.00	1.07
*D*_*m*_	1.54	1.47	1.93	1.36	1.86	2.00	2.47	2.29	2.16	2.11
$E_{glob}^{t}$	0.11	0.14	0.07	0.11	0.46	0.41	0.41	0.36	0.44	0.34
$E_{glob}^{m}$	0.65	0.76	0.62	0.75	0.65	0.49	0.52	0.44	0.65	0.45
$E_{loc}^{t}$	5.38×^−03^	0.00e+00	3.17e-02	2.00e-02	6.97e-01	4.19e-01	4.50e-01	3.66e-01	2.64e-01	2.80e-01
$E_{loc}^{m}$	9.60e-05	0.00e+00	1.90e-03	8.83e-04	1.36e-01	7.62e-02	6.03e-02	8.09e-02	5.61e-02	4.81e-02
*C*_*t*_	1.64e-02	2.56e-02	5.07e-03	7.98e-03	4.71e-02	7.56e-02	6.42e-02	6.11e-02	7.83e-02	1.33e-02
*C*_*m*_	1.89e-03	3.19e-03	3.63e-04	7.96e-04	3.46e-02	5.53e-02	5.58e-02	5.43e-02	6.27e-02	1.15e-02
*C*_*t*_/*E*_*t*_	1.50e-01	1.80e-01	6.00e-02	7.00e-02	1.00e-01	1.80e-01	1.50e-01	1.60e-01	1.70e-01	3.00e-02
*C*_*m*_/*E*_*m*_	0.00e+00	0.00e+00	0.00e+00	0.00e+00	5.00e-02	1.10e-01	1.00e-01	1.20e-01	9.00e-02	2.00e-02

Datasets statistics of subways, the US airline and city-based online social networks: number of nodes *n* and edges *m* of the graphs, maximum degree *k*_*max*_ and average node degree ⟨*k*⟩, standard deviation of the degree *σ*_*k*_, assortativity mixing by degree *ρ*, physical, topological and metrical diameter *D*, global and local efficiency *E*_*glob*_, *E*_*loc*_, costs and *C*/*E* property (defined as the ratio between cost and global efficiency). Both *topological* and *metrical* versions are calculated of the latter three indicators.

Among these datasets, subway networks are neither scale-free nor small-world because the diameter *D*_*t*_ does not scale as log(*n*), the average shortest path *L* is high, the clustering coefficient is low (like in random networks) and efficiency is also low (see Table 1 and classification [43]). On average, these networks have low degree nodes, i.e. the majority of stations are not interchange points where users can switch to other lines. The maximum degree is 4, or 8 for the biggest subways (they can not be considered *hubs* as in scale-free networks though) and they are assortative or eventually uncorrelated. However, by considering the weighted version of the networks, we found that subways are very efficient both locally and globally ($E_{glob}^{m}>0.65$). This observation is also confirmed by previous studies [35] [36] in which the authors tested the small world property on the Boston and worldwide subways.

**Fig 12:**
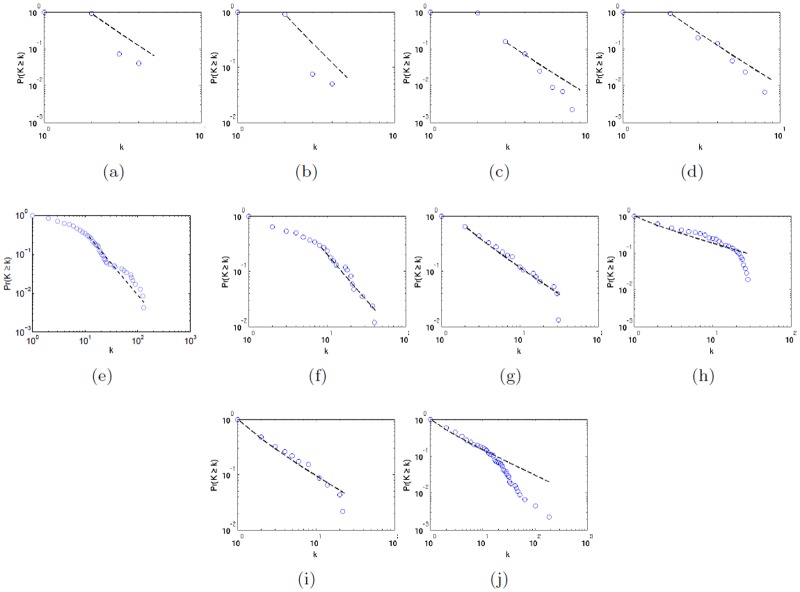
*P*_*cum*_ distribution of subways, transportation and social networks. The log-log plots of the cumulative degree distributions *P*_*cum*_(*k*) of subways (Boston, Milan, New York, Paris, a to d), the US airline (e) and city-based online social networks (letter f to j) of Italy, Australia, The Netherlands, India and the United Kingdom. The distributions are characterized by exponents *γ* of *P*(*k*) ∼ *k* ^−*γ*^ that is one plus the slope of *P*_*cum*_(*k*) (in a log-log plot), i.e. *γ* = 1 + *γ*_*cum*_. The coefficient is *γ* = 3.5 for subways networks, 2.6 for the US airline, 1.85 for Indian city-based online social network, 1.68 for the United Kingdom, 2.61 for Italy, 1.94 for Australia and 1.61 for the Netherlands. The coefficients for subways might not be precise due to the small dimension of the networks.

On the other hand, the city-based online social networks and the US airline transportation networks have a different connection pattern. We found that two randomly nodes are connected by means of less than ten edges and the clustering coefficient is rather high. *k*_*max*_ and *σ*_*k*_ are high compared to subway networks and the degree distributions all displays long right tails (see Fig. 12 letter e to j) that is evidence for the presence of hubs. Indeed, high efficiency and low diameter are detected. On average, high degree nodes tend to be connected to low degree nodes (*ρ* is always negative) like in technological, neural and protein-to-protein interactions networks [31].

## Discussion

In this section, we report on the results obtained by applying the micro-macro analysis to real world and artificial networks and we show how this novel approach based on modifications of the spatial axis on complex systems is effectively a robust tool that precisely describes networks at different detail levels.

In all the experiments presented in this paper, we randomly shift 10^4^ times the position of the square boxes to limit the bias in the grid displacement (the entire set of boxes will be shifted, not the single boxes individually, see the previous section), and we eventually take averaged results.

The telescopic process creates a micro-macro spectrum $S_{G}=\{G_{1},G_{2},\ldots ,G_{k}\}$ where *G*_*i*_ = *t*(*G*, *f*_*i*_), *i* = 1, … *k*. We selected *k* = 100 as the granularity of the telescopic spectrum, therefore the fuzziness will be (linearly) increased by 0.01 units at each step.

In the plots that follow, for the sake of clarity, we decided not to consider the most abstracted network (at *f* = 1) since it contains only one node and consequently the metrics will get trivial results. Furthermore, some plots contain relative quantities. This means that the value obtained with fuzziness *f*, say *v*_*f*_, will be divided by *v*_*f* = 0_, that is, the value obtained with no abstraction at all. This helps to depict the increase or decrease relative to the baseline.

**Fig 13:**
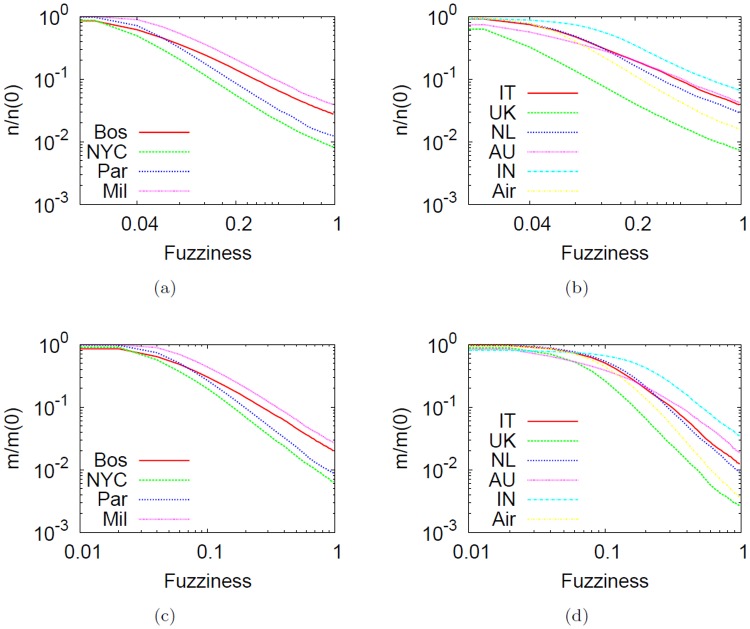
Number of collapsed nodes and edges as a function of *f* in log-log axes. Number of collapsed nodes *n* and edges *m* as a function of *f* in log-log axes. The values are normalized by the baseline values *n*(0) and *m*(0) respectively, obtained at *f* = 0 (i.e., no abstraction applied). The leftmost panels refer to subway networks whereas the rightmost refer to city-based online social networks and the US airline network. The decrease of *n* and *m* is clearly exponential, even though the rate is influenced by many factors like network size and node positions.

Fig. 13 shows how nodes and edges are merged together as a function of fuzziness *f*. It is interesting to note that the overall behavior of collapsing nodes and edges is similar over the same type of network (transportation and city-based online social networks). However, the rate with which vertices and links are merged depends on several factors such as the size of the system (instead of the history), the physical position and the structure of the network itself. Bigger networks, for instance New York or Paris have a faster merging rate.

**Fig 14:**
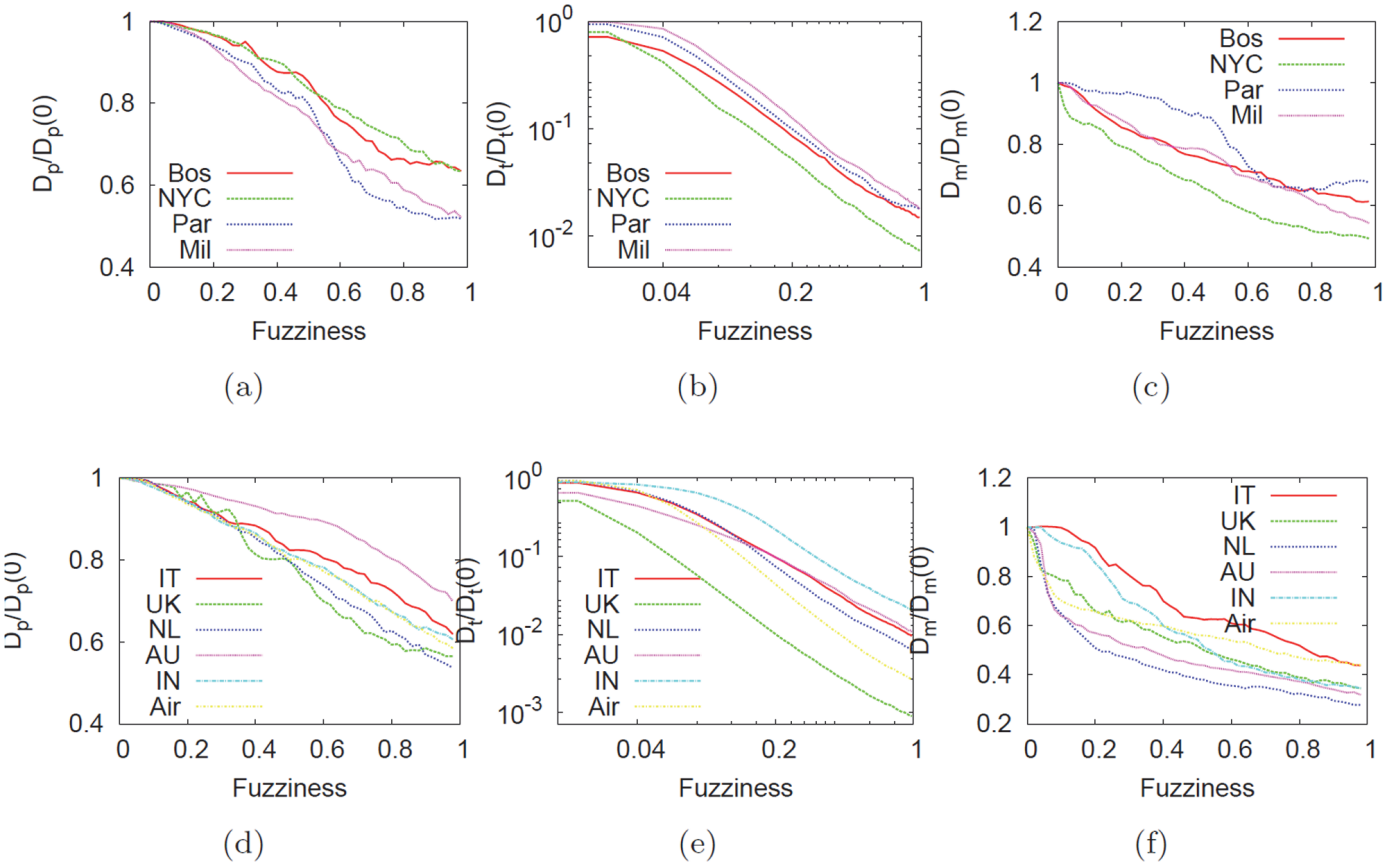
Effect of the telescopic abstraction on the diameter as a function of *f*. Effect of the telescopic abstraction for the physical *D*_*p*_, topological *D*_*t*_ and metrical *D*_*m*_ diameter as a function of fuzziness *f*. All the values were normalized by the baseline values at *f* = 0 (i.e., no abstraction is applied). The top panels contain results of subways, the bottom ones of city-based online social networks and the US airline network.

Fig. 14 shows how diameters metrics vary as a function of fuzziness *f*. The three versions of these statistical quantities accounts for three different characteristic of the networks: (i) the maximum physical extension of nodes in a 1 × 1 unit square box, (ii) the maximum topological extension on the shortest path and (iii) the maximum metrical extension on the shortest weighted path. The first one, as expected, decreases linearly, mainly because of the linear increase of the fuzziness. The second one decreases exponentially and this is evidence that the telescopic process creates the right shortcuts links that decrease faster the diameter.

**Fig 15:**
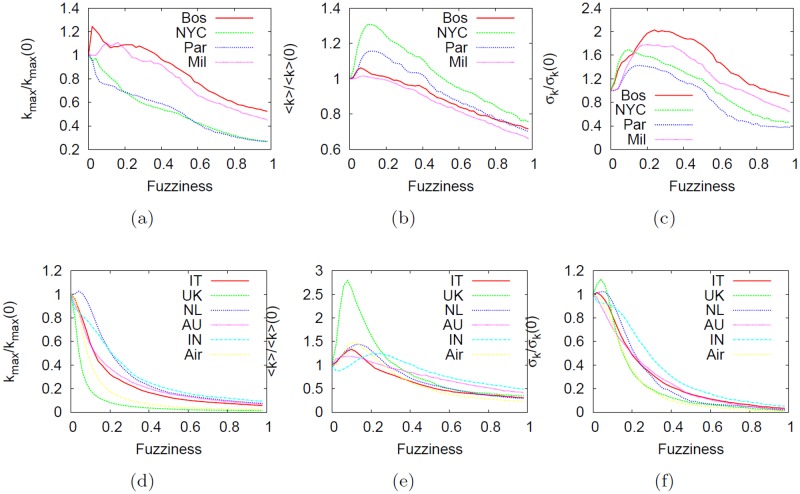
Effect of the telescopic analysis on the degree. Effect of the telescopic analysis on the degree: maximum degree *k*_*max*_ (leftmost column), mean degree ⟨*k*⟩ (center column) and standard deviation *σ*_*k*_ (rightmost column) for subways (top panels), the US airline and city-based online social networks (bottom panels). All values were normalized relatively to the baseline value at *f* = 0 (where no abstraction is applied). The explanation of the results obtained is not so trivial. In general, the degree properties of the networks will be drastically modified as fuzziness increases. The degree tend to decrease linearly in subways whereas in airline and social-based networks the telescopic effect results in an exponential decrease.

Fig. 15 shows the effect of the telescopic abstraction on *k*_*max*_, ⟨*k*⟩ and standard deviation of the degree *σ*_*k*_. The explanation of the observed behavior is not so trivial even though a couple of observations can be made. First, we note a clear distinction on results between the two types of networks (first and second row). For instance, in subway networks, *k*_*max*_ decrease almost linearly and a joint observation that takes into account both ⟨*k*⟩ and *σ*_*k*_ suggests that telescopic analysis triggers an increase of the average degree (even though this is not necessarily evidence that in some part of the telescopic spectrum, the analysis produces networks with hubs). Conversely, in city-based online social networks and the US airline network (bottom panels), the effect of abstraction on the degree is more prominent. Where in subways the decrease on relative *k*_*max*_ was almost linear, in these networks the rate with which the maximum degree decreases is exponential. This is mostly caused by the presence of hubs that will be collapsed almost immediately as fuzziness *f* increases.

**Fig 16:**
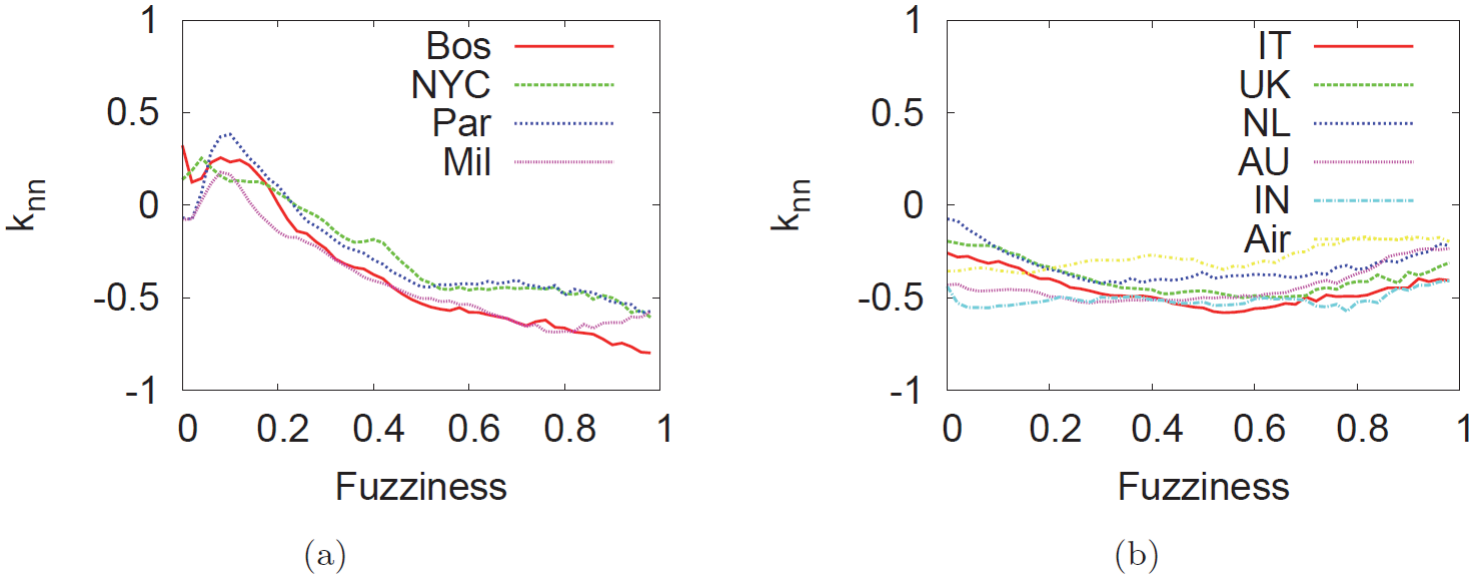
Impact of the telescopic analysis on the degree correlations *ρ*. Impact of the telescopic analysis on degree correlations *ρ* as a function of *f* for subway networks (left panel), the US airline and city-based online social networks (right panel). It is worth noting that the telescopic process yields disassortative networks regardless of the network. This means that in subways, the topological structure will be drastically changed whereas in the other networks the degree correlation tends to remain stable (at least will have the same sign).

Degree correlations *k*_*nn*_ on (unweighted) networks measure the level of interdependence between nodes. From Table 1 we identified two different connection pattern as we consider subways or city-based online social network and the US airline network. The first class of networks is neutral whereas the second one is negatively correlated (that is, nodes with high degree link to small degree nodes). Fig. 16 summarize the degree correlations in the telescopic spectrum. We note that the abstraction process yields disassortative networks at high values of fuzziness regardless of the system considered. As a consequence, the initial topological structure of subway networks will be significantly changed toward a completely different configuration whereas in city-based online social networks and the airline network remain relatively stable in the entire spectrum (at least they remain disassortative).

**Fig 17:**
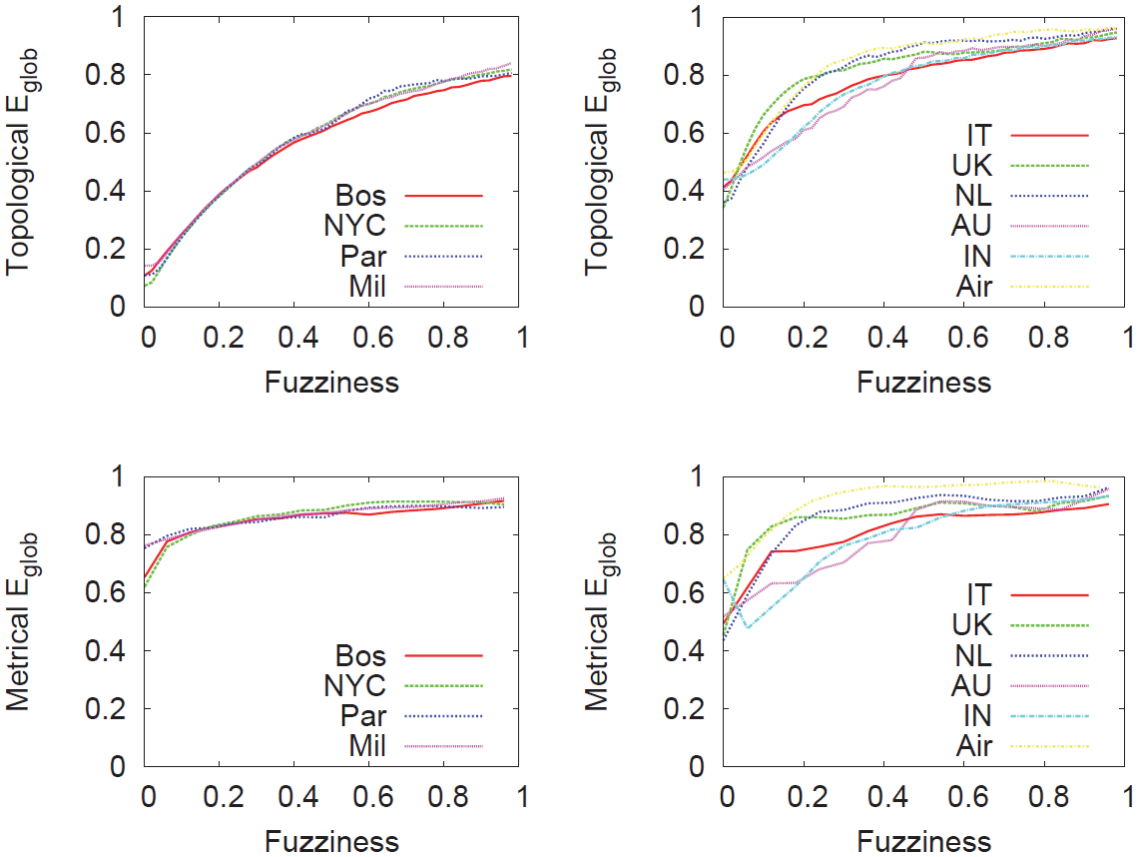
Effect of the telescopic process on *E*_*glob*_. Effect of the telescopic process on subways (leftmost column), the US airline and city-based online social networks (rightmost column) as a function of *f*. The statistical properties considered in these panels are topological and metrical *E*_*glob*_. The abstraction process does not preserves the topological *E*_*glob*_ (top panels) while varying *f*. In particular, regardless of the network considered, the networks viewed at macro level are simpler and more efficient compared to micro view. Conversely, the situation is slightly different for metrical *E*_*glob*_ (bottom panels). In this case, the connection pattern of the system considered alters significantly the outcome of the abstraction process. In fact, we detected that the structure of subway networks allow a good preservation of the metrical efficiency in the spectrum whereas in city-based online social networks this feature is absent.

Fig. 17 shows how global efficiency *E*_*glob*_ is influenced by the granularity level with which a network is described. In particular, metrical and topological versions are considered. One of the aims of this study is to verify whether the detail scale with which networks are described affects network efficiency. Different observations can be made for topological and metrical quantities. Firstly, we clearly note (top panels) that the efficiency is strongly influenced by the current fuzziness value, regardless of the networks considered. In particular, at micro level (that is, when the network structure is highly detailed) the efficiency is smaller compared to the macro level. This is an interesting element suggesting that every shift in the abstraction process is effectively a useful methodology to simplify a system (in fact the number of nodes and edges decrease, see Fig. 13) by eventually selecting the substructure of the network that works best and that is most efficient. One element that distinguishes the two different types of networks is the connection pattern, reflected at micro level. We clearly note that as the fuzziness increases the two classes of networks tend to gradually be similar, smoothing away the initial differences.

Secondly, we notice how metrical *E*_*glob*_ of subways networks (bottom left panel) is reasonably stable over the telescopic spectrum, meaning that their metrical features will be preserved during the abstraction process. However, this finding holds only in exponential networks like subways where the metrical element plays an important role during network creation and evolution. Conversely, in networks embedded in Euclidean space but where physical constraints on edges are relaxed (like in the US airline and city-based online social networks, right panels) the *E*_*glob*_ (both topological and metrical) is not universal in the spectrum and again strongly depends on fuzziness.

Generally speaking, this finding is evidence that unraveled by micro-macro analysis, all the results of analysis on scale-free small-world networks to date refer only to a specific resolution level (fuzziness) and therefore depend on the level of detail: so, some of those results could be just an effect observable at a certain scale, whereas the behavior of the complex system at a lower micro level (or at a higher macro level of abstraction) could be completely different.

*E*_*glob*_ is a quantity that accounts for the global system flow of information along the paths of the networks. Conversely, with the formalization of the local efficiency *E*_*loc*_ (see the subsection Statistical Properties), it is possible to detect how efficiently a system exchange information in the node neighborhoods.

**Fig 18:**
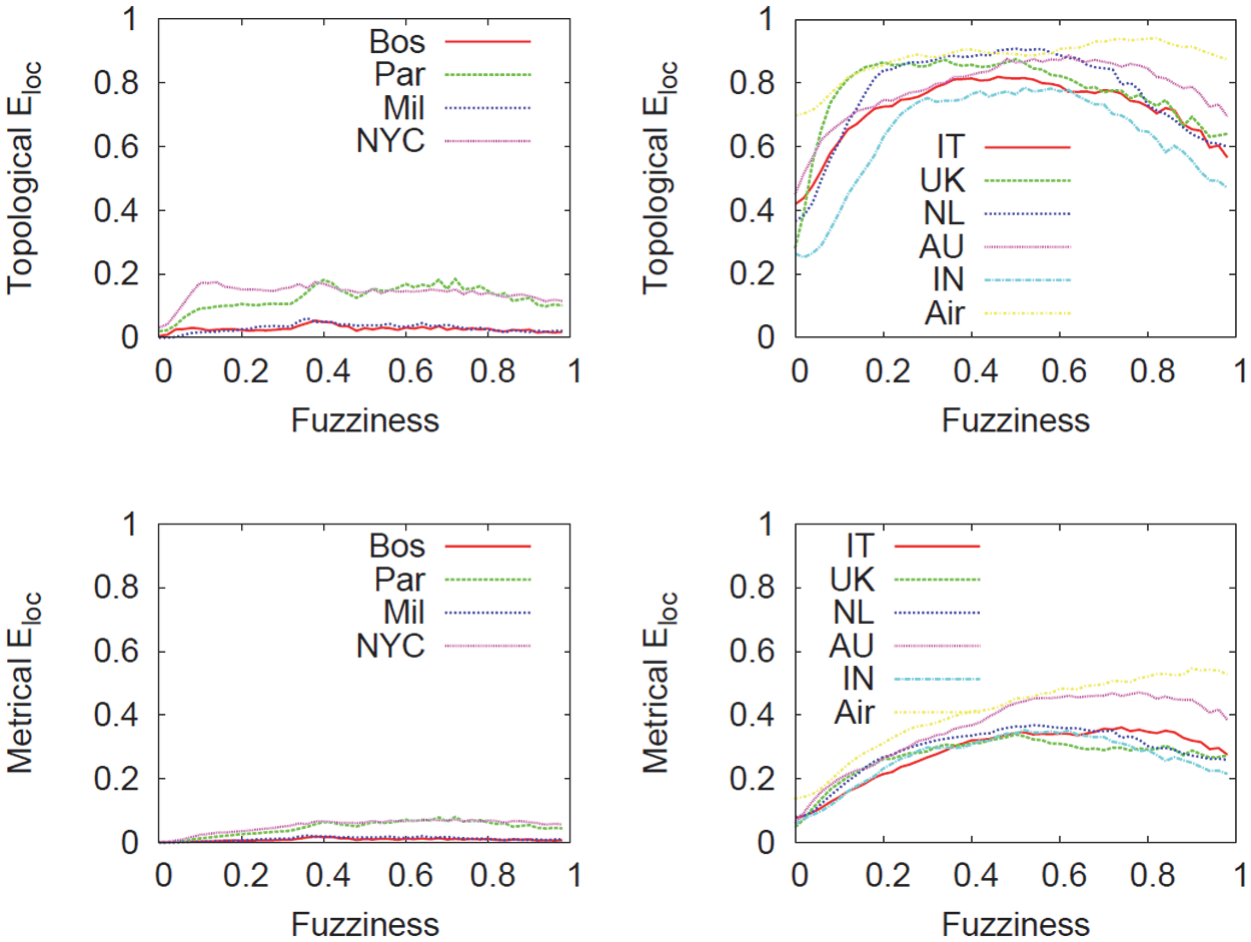
Effect of the telescopic analysis on *E*_*loc*_. Effect of the telescopic analysis on topological and metrical *E*_*loc*_ as a function of *f* in subways (leftmost panels), the US airline and city-based online social networks (rightmost panels). The left most panels show that *E*_*loc*_ is almost stable in the spectrum meaning that the local properties of the subway networks are preserved by the analysis. However, in networks with heterogeneous topological structure, the telescopic process will further increase *E*_*loc*_ resulting in the creation of systems that are densely connected at local level.

Fig. 18 shows how the telescopic analysis affects local efficiency as a *f* increases. At micro scale, we distinguish a completely different local connection pattern between the two types of networks (when no abstraction is applied). In fact, we note that subways are locally poorly connected because of the intrinsic physical and economic constraints that govern the growth. The US airline and city-based online social networks, that are almost free from constraints (at least in the way nodes are linked), will have more redundant edges that increase local efficiency.

We observe again that the overall behavior of the considered metric is strongly influenced by the type of dataset involved. The main difference between the two types is not in the order of magnitude with which *E*_*loc*_ increases (in fact in both cases the quantity will raise) but instead in the detected behavior over the spectrum. We noted that *E*_*loc*_ is much more stable in subway networks compared to airline and city-based online social networks. This is probably caused by the characteristic of redundancy in the topological structure that makes *E*_*loc*_ more variable in the telescopic spectrum.

**Fig 19:**
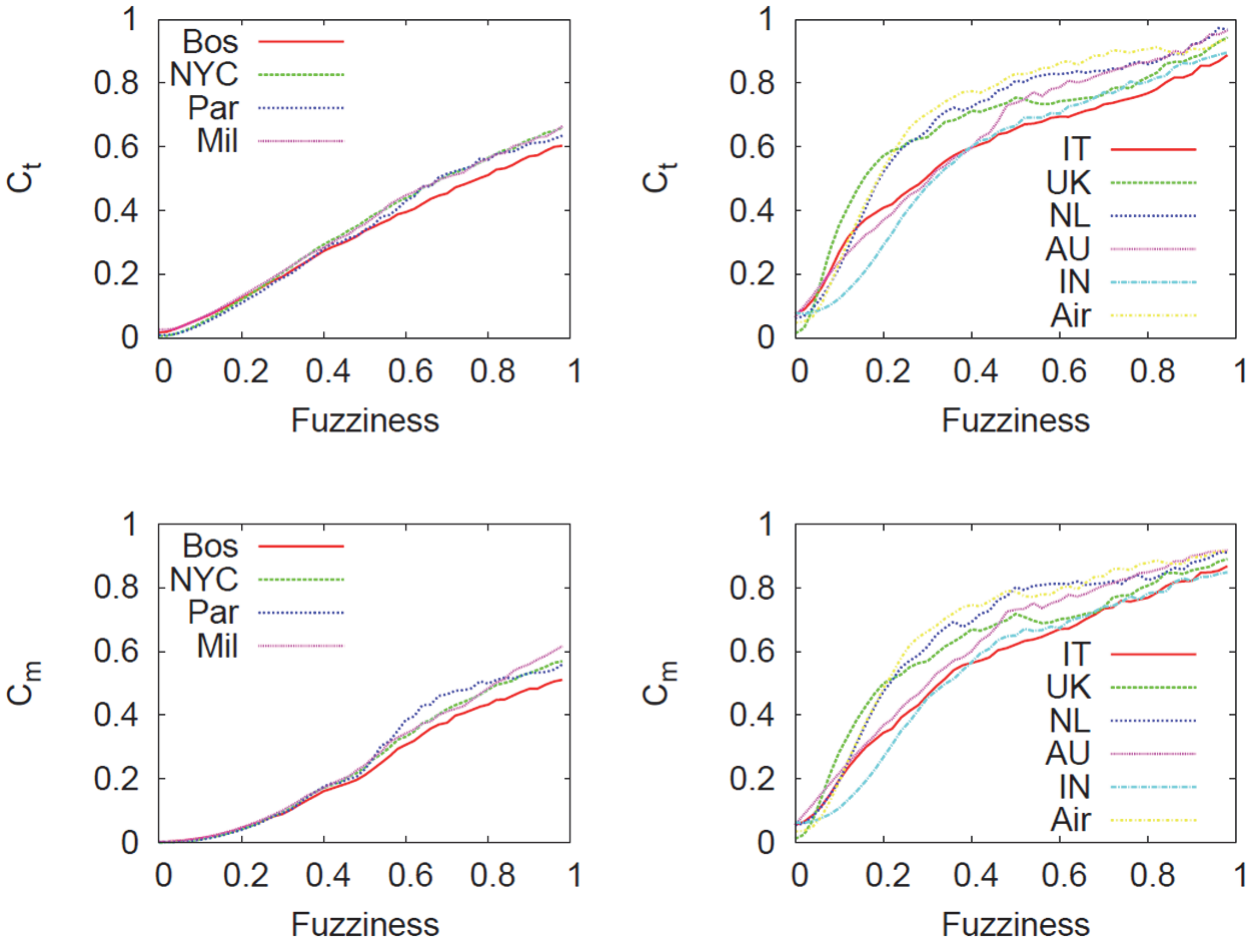
Effect of the telescopic analysis on *c*_*t*_ and *c*_*m*_. Effect of the telescopic analysis on topological and metrical cost (*c*_*t*_ and *c*_*m*_) as a function of *f* for subways (leftmost column), the US airline and city-based online social networks (rightmost column). We note that our coarse graining process produces networks more expensive than detailed ones. This effect might be caused by the creation of redundant structures in macro level systems so that the whole cost will be higher. Even though both curves are positively correlated to *f*, the slope in subways networks is smaller compared to city-based online social networks. To verify whether this effect is not trivially caused by a low efficiency value, we will consider *C*/*E*_*glob*_ index (see Fig. 20).

Even though local and global quantities are essential to characterize a network, the cost is another factor that has to be considered in order to get a better understanding of the entire system. Fig. 19 reveals how metrical and topological costs behave along the telescopic spectrum. Regardless of the networks, we clearly see that as fuzziness *f* increases, the overall network cost will raise.

Although it seems counterintuitive because abstracted, i.e., simple networks should be cheap, it is an expected effect because (as Figs. 17 and 18 show) these are also very efficient and as such, very expensive. All the curves are monotonically increasing functions, but subways result in smaller increase compared to city-based online social networks. This represents evidence that these networks have an economic inborn principle that is maintained also during the abstraction process.

**Fig 20:**
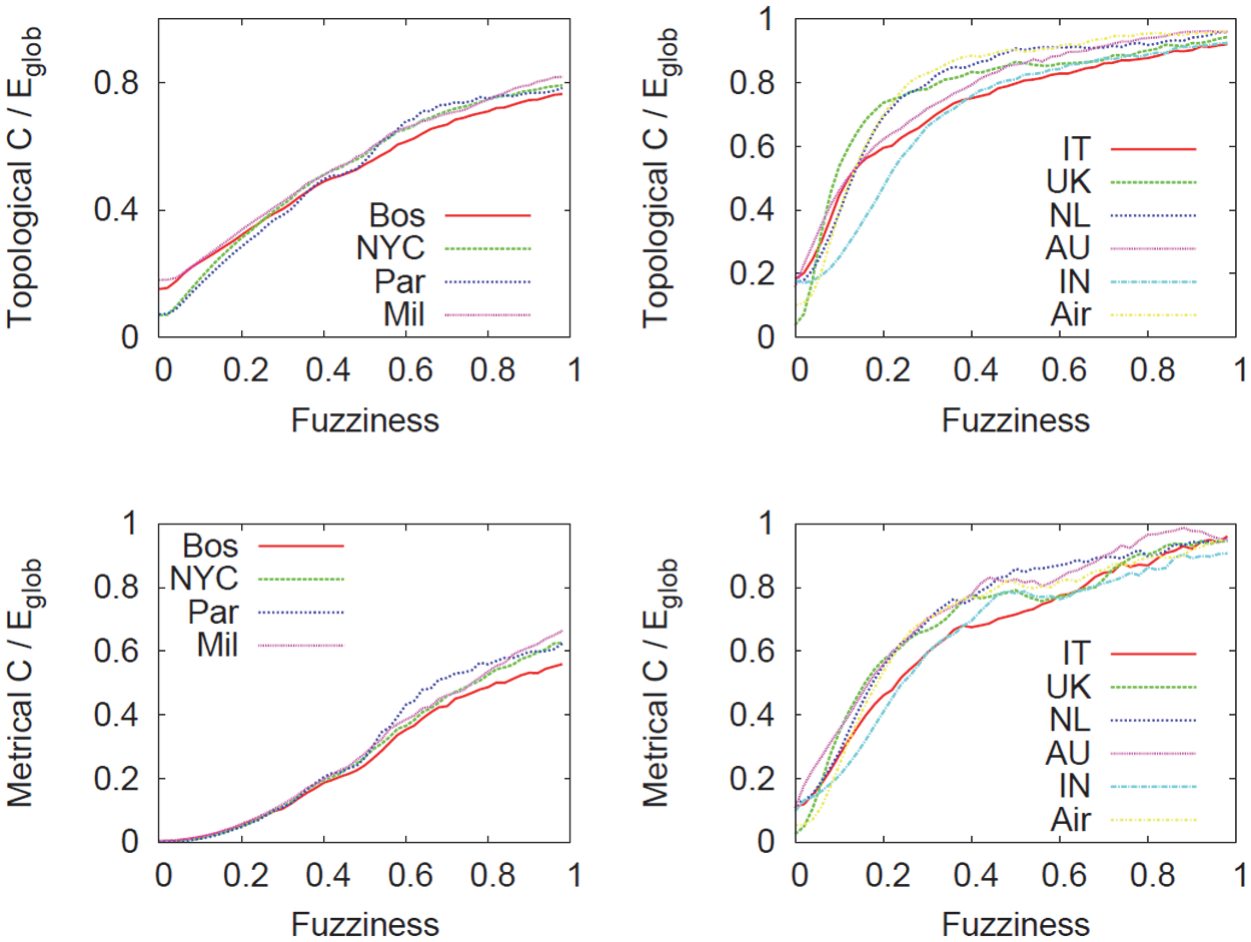
Effect of the telescopic analysis on *C*/*E*_*glob*_. Effect of the telescopic analysis on topological and metrical normalized cost over efficiency for subways (leftmost column), the US airline and city-based online social networks (rightmost column). By dividing the cost of the networks by the global efficiency (that ranges between 0 and 1), we verified that subway networks are cheaper as well as very efficient, more than city based online social networks. This is evidence that subway network have an economic inborn principle that is maintained during the telescopic abstraction process.

Networks are defined as economic [26] when they have low cost and high efficiency, i.e. whenever the ratio *C*/*E* tends to zero. Fig. 20 shows how this variable changes in the telescopic spectrum. We clearly see that detailed networks have a better cost/benefit ratio than coarse-grained ones.

**Fig 21:**
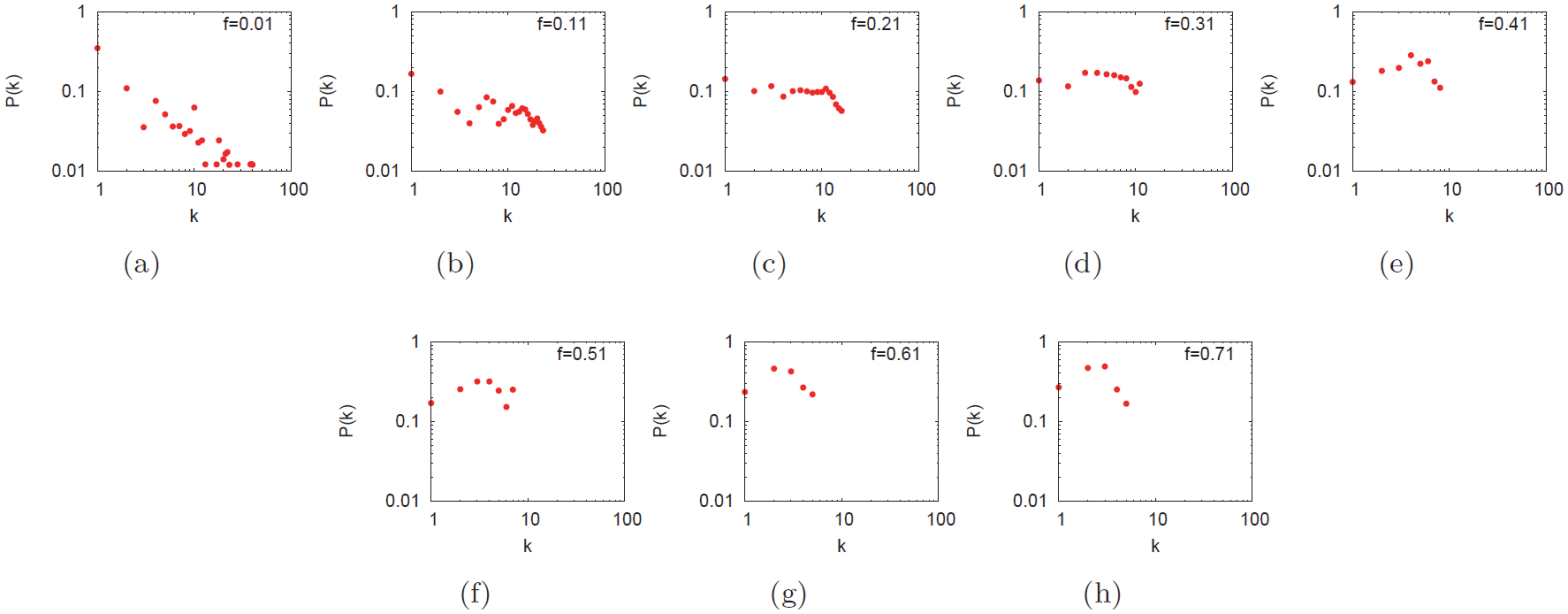
Effect of the abstraction process on the degree distribution *P*(*k*). Effect of the abstraction process on the degree distribution *P*(*k*) for increasing values of fuzziness *f* for the Netherlands city-based online social network. We detected that the behavior starts from a small world scale-free configuration and is ideally maintained for *f* < 0.11. When *f* increases, it changes to uniform and finally to random (when *f* is maximum).

Fig. 21 shows how the degree distribution *P*(*k*) changes by increasing the fuzziness by 0.1 units at each step. We find that when decreasing the detail level, networks tend to lose their original topological structure and every node is likely to have the same degree. Therefore, hubs disappear and they became like low degree nodes. This is an expected result because it follows from the definition of network abstraction.

### Conclusions

In this paper, we have introduced a novel network analysis that we called micro-macro. This new framework consists of (meta-)studying the important informational axis along with a complex network can be seen: the level of detail.

The so-called telescopic scaler, inspired from the human eyes capability to distinguish two points when placed at some distance from a point of view, was devoted to propose a new method that arbitrarily models networks under different levels of abstraction. Its importance stems from the ability of changing the spatial coordinates and connectivity of the nodes according to some predefined rules. Doing so, we were able to understand what happens to the most important statistical network properties not only when the network detail is high (at micro level) or low (at macro level), but also in between these two extremes. At this point, we were concerned to answer a set of questions such as: which properties are safe to consider after abstracting a network? Which topological structures better preserve system attributes? Are the results of static analysis possibly incomplete because they strongly rely on the detail level with which a network is constructed?

Our experiments were focused on networks that are embedded in the space, whose evolution is constantly shaped by the surrounding environment. We considered rapid transportation networks (such as subways and airlines) and city-based online social networks. An important finding suggests that complex networks, when observed at finer or coarse-grained level of detail, exhibit statistical features that in many cases are different, meaning that networks characteristics are not stable under the telescopic (or abstraction) process. Because of that, many networks researches are confined to describe only one of all the possible configurations a network could take, showing results that might not be valid for the entire grained spectrum.

The analysis of the full micro-macro spectrum also helps to shed light on how much all the properties of a complex system depend on the level of detail, showing their transition patterns and their relative stability, and on how much previous results on coarse-graining and the fractal dimension of networks do apply to real metric networks as well.

Last, but not least, we note how micro-macro analysis, and the specific telescopic approach used here, have anyway a more general scope rather than being just limited to euclidean-space 2d environments. Micro-macro analysis is a general concept, and relies on a distance space that can generally define relations of proximity between objects of a system (see for instance [44]). From this geometry of interactions we can then define the appropriate notions of fuzzy abstractions, for instance via a suitable generalization of the telescopic algorithm or some other variants. One possible variant is for instance the 3d telescopic scaler, obtained by the obvious generalization of the 2d telescopic scaler to three dimensions, which allows to tackle micro-macro analysis on crucially important systems like brain networks without artificially lowering their dimensionality. Therefore, micro-macro analysis accounts for all contexts where an observability scale matters, and the case studies analyzed in this paper (transportation and online social systems) are an interesting, but not at all comprehensive, starting sample to better understand the phenomena that occur when the level of detail varies.
